# Corrigendum: Characterization of the Interactive Effects of Labile and Recalcitrant Organic Matter on Microbial Growth and Metabolism

**DOI:** 10.3389/fmicb.2021.682681

**Published:** 2021-07-12

**Authors:** Lauren N. M. Quigley, Abigail Edwards, Andrew D. Steen, Alison Buchan

**Affiliations:** ^1^Department of Microbiology, The University of Tennessee, Knoxville, Knoxville, TN, United States; ^2^Department of Earth and Planetary Sciences, The University of Tennessee, Knoxville, Knoxville, TN, United States

**Keywords:** interactive effects, terrestrially derived, dissolved organic matter, Roseobacter, species-specificity, community interactions

In the original article, there was an error. Incorrect units were listed for some components of our growth medium.

A correction has been made to **Materials and Methods**, subsection **Strains, Media, and Growth Conditions**, paragraph 1. The corrected paragraph appears below.

“*Sagittula stellata* sp. E-37, *Citreicella* sp. SE45, *Phaeobacter* sp. Y4I, *Roseovarius nubinhibens* ISM, *Sulfitobacter* sp. EE-36, and *Sulfitobacter* sp. NAS-14.1 were routinely grown on an aromatic basal medium (ABM) containing per liter 8.7 mM KCl, 8.7 mM CaCl_2_, 43.5 mM MgSO_4_, and 174 mM NaCl with 225 μM K_2_HPO_4_, 13.35 mM NH_4_Cl, 71 mM Tris-HCl (pH 7.5), 68 μM Fe-EDTA, trace metals (7.8492 mM Nitroloacetic acid, 0.5325 mM MnSO_4_*H_2_O, 0.4203 mM CoCl_2_*6H_2_O, 0.3478 mM ZnSO_4_*7H_2_O, 0.0376 mM CuSO_4_, 0.1052 mM NiCl_2_*6H_2_0, 1.1565 mM Na_2_SeO_3_, 0.4134 mM Na_2_MoO_4_*2H_2_O, 0.3259 mM Na_2_WO_4_*2H_2_O, 0.2463 mM Na_2_SiO_3_*9H_2_O) and trace vitamins (0.0020% vitamin H [Biotin)], 0.0020% folic acid, 0.0100% pyridoxine-HCl (B6), 0.0050% riboflavin (B2), 0.0050% thimaine (B1), 0.0050% nicotinc acid, 0.0050% pantothenic acid (B5), 0.0001% cyanocobalamin (B12), 0.0050% *p*-aminobenzoic acid). These strains were routinely passaged on ABM containing 10 mM sodium acetate. Four of these strains (E-37, SE45, Y4I, and EE-36) were isolated from Southeastern US coastal waters, while NAS-14.1 was isolated from North Atlantic off-shore waters and ISM from the Caribbean Sea (Buchan et al., 2000; Cude et al., 2012). The bacteria were routinely cultured at 30°C, shaking, in the dark. This temperature condition is nominally representative of Southeastern US salt marshes which are tidally influenced and where average water temperatures are close to 30°C from June through September (The Southeast Regional Climate Center, University of North Carolina, Chapel Hill, NC). Suwannee River natural organic matter (NOM), obtained from the International Humic Substance Society (IHSS, St. Paul, MN) was used as a representative t-DOM. This material is a discipline standard for natural organic matter (Her et al., 2003). Incubations occurred in the dark as the aromatic moieties in NOM are sensitive to photodegradation. NOM is provided in lyophilized form from IHSS and was suspended in Milli-Q water and 0.22 μm filter-sterilized prior to addition to the medium. NOM was held at a constant concentration of 2 mM-C for all experiments. ^13^C NMR estimates of carbon distribution provided by IHSS show that Suwannee NOM comprised of roughly 25% aromatic residues.”

The authors apologize for this error and state that this does not change the scientific conclusions of the article in any way.

The original article has been updated.
