# Supplementary material for: Characterization of the Interactive Effects of Labile and Recalcitrant Organic Matter on Microbial Growth and Metabolism
Source: Front Microbiol. 2019 Mar 19;10:493. doi: 10.3389/fmicb.2019.00493 (PMC6433851; doi:10.3389/fmicb.2019.00493)
Supplement: Supplementary file 1 [file Data_Sheet_1.PDF]

Supplemental Tables (Quigley et al)

Supplemental Table S1. ANOVA Tables for each three-way ANOVA performed for SE45 cell density by day.

Day	ANOVA Table					
1		Df	Sum Sq	Mean Sq	F value	Pr(>F)
	Treatment	1	0.14	0.145	3.866	0.05143
	Concentration	3	46.41	15.470	413.565	< 2e-16
	Carbon	3	0.90	0.300	8.011	6.19e-05
	Treatment:Concentration	3	1.75	0.582	15.556	1.10e-08
	Treatment:Carbon	3	0.46	0.154	4.110	0.00804
	Concentration:Carbon	9	3.49	0.388	10.372	6.39e-12
	Treatment:Concentration:Carbon	9	2.21	0.246	6.566	1.14e-07
	Residuals	128	4.79	0.037		
2		Df	Sum Sq	Mean Sq	F value	Pr(>F)
	Treatment	1	0.40	0.397	14.620	0.000211
	Concentration	3	51.95	17.318	638.473	< 2e-16
	Carbon	3	2.15	0.715	26.378	3.75e-13
	Treatment:Concentration	3	0.96	0.320	11.812	7.92e-07
	Treatment:Carbon	3	0.03	0.010	0.351	0.788349
	Concentration:Carbon	9	3.22	0.358	13.203	1.90e-14
	Treatment:Concentration:Carbon	9	0.31	0.034	1.271	0.259552
	Residuals	119	3.23	0.027		
4		Df	Sum Sq	Mean Sq	F value	Pr(>F)
	Treatment	1	0.67	0.666	28.833	3.70e-07
	Concentration	3	32.28	10.760	465.940	< 2e-16
	Carbon	3	0.75	0.250	10.836	2.23e-06
	Treatment:Concentration	3	0.59	0.196	8.500	3.50e-05
	Treatment:Carbon	3	0.47	0.156	6.738	0.000299
	Concentration:Carbon	9	5.08	0.564	24.423	< 2e-16
	Treatment:Concentration:Carbon	9	0.70	0.078	3.361	0.001020
	Residuals	125	2.89	0.023		
7		Df	Sum Sq	Mean Sq	F value	Pr(>F)
	Treatment	1	0.003	0.003	0.100	0.752828
	Concentration	3	18.786	6.262	210.239	< 2e-16
	Carbon	3	0.501	0.167	5.606	0.001219
	Treatment:Concentration	3	0.387	0.129	4.332	0.006084
	Treatment:Carbon	3	1.031	0.344	11.539	9.82e-07
	Concentration:Carbon	9	3.736	0.415	13.939	2.23e-15
	Treatment:Concentration:Carbon	9	1.001	0.111	3.734	0.000347
	Residuals	126	3.753	0.030		
10		Df	Sum Sq	Mean Sq	F value	Pr(>F)
	Treatment	1	0.020	0.020	0.643	0.424108
	Concentration	3	15.930	5.310	172.504	< 2e-16
	Carbon	3	0.643	0.214	6.959	0.000232
	Treatment:Concentration	3	1.206	0.402	13.060	1.88e-07
	Treatment:Carbon	3	0.399	0.133	4.321	0.006221
	Concentration:Carbon	9	1.131	0.126	4.083	0.000133
	Treatment:Concentration:Carbon	9	0.843	0.094	3.042	0.002571
	Residuals	122	3.755	0.031		
14		Df	Sum Sq	Mean Sq	F value	Pr(>F)
	Treatment	1	0.217	0.2170	4.739	0.031419
	Concentration	3	9.323	3.1078	67.872	< 2e-16
	Carbon	3	2.528	0.8427	18.405	6.48e-10
	Treatment:Concentration	3	1.183	0.3942	8.609	3.14e-05
	Treatment:Carbon	3	0.192	0.0638	1.394	0.247777
	Concentration:Carbon	9	1.714	0.1904	4.158	0.000107
	Treatment:Concentration:Carbon	9	0.420	0.0467	1.020	0.428257
	Residuals	122	5.586	0.0458		

Supplemental Table S2. ANOVA Tables for each three-way ANOVA performed for E-37 cell density by day.

Day	ANOVA table	Df	Sum Sq	Mean Sq	F value	Pr(>F)
1	Treatment	1	0.004	0.004	0.269	0.60469
	Concentration	3	28.549	9.516	649.373	< 2e-16
	Carbon	3	1.715	0.572	38.999	< 2e-16
	Treatment:Concentration	3	0.231	0.077	5.257	0.00192
	Treatment:Carbon	3	0.620	0.207	14.102	6.09e-08
	Concentration:Carbon	9	3.846	0.427	29.159	< 2e-16
	Treatment:Concentration:Carbon	9	1.185	0.132	8.983	2.67e-10
	Residuals	121	1.773	0.015		
2	Treatment	1	0.017	0.017	0.466	0.49587
	Concentration	3	15.543	5.181	139.414	< 2e-16
	Carbon	3	4.327	1.442	38.814	< 2e-16
	Treatment:Concentration	3	0.419	0.140	3.755	0.01266
	Treatment:Carbon	3	0.633	0.211	5.682	0.00111
	Concentration:Carbon	9	2.536	0.282	7.581	7.93e-09
	Treatment:Concentration:Carbon	9	0.412	0.046	1.233	0.28075
	Residuals	126	4.682	0.037		
4	Treatment	1	0.190	0.190	2.547	0.11314
	Concentration	3	11.964	3.988	53.593	< 2e-16
	Carbon	3	5.806	1.935	26.010	4.70e-13
	Treatment:Concentration	3	0.263	0.088	1.177	0.32142
	Treatment:Carbon	3	1.062	0.354	4.758	0.00359
	Concentration:Carbon	9	3.548	0.394	5.297	4.38e-06
	Treatment:Concentration:Carbon	9	2.056	0.228	3.070	0.00238
	Residuals	121	9.004	0.074		
7	Treatment	1	2.255	2.255	57.171	7.73e-12
	Concentration	3	0.294	0.098	2.486	0.0637
	Carbon	3	11.767	3.922	99.457	< 2e-16
	Treatment:Concentration	3	0.142	0.047	1.200	0.3126
	Treatment:Carbon	3	1.493	0.498	12.618	2.97e-07
	Concentration:Carbon	9	5.829	0.648	16.422	< 2e-16
	Treatment:Concentration:Carbon	9	2.880	0.320	8.113	2.13e-09
	Residuals	124	4.890	0.039		
10	Treatment	1	0.006	0.0056	0.101	0.7513
	Concentration	3	5.987	1.9957	35.790	< 2e-16
	Carbon	3	9.430	3.1432	56.370	< 2e-16
	Treatment:Concentration	3	1.624	0.5414	9.709	8.91e-06
	Treatment:Carbon	3	2.209	0.7364	13.206	1.71e-07
	Concentration:Carbon	9	4.080	0.4534	8.131	2.56e-09
	Treatment:Concentration:Carbon	9	1.112	0.1235	2.216	0.0255
	Residuals	118	6.580	0.0558		
14	Treatment	1	0.110	0.110	2.995	0.0861
	Concentration	3	20.854	6.951	189.836	< 2e-16
	Carbon	3	3.376	1.125	30.733	7.69e-15
	Treatment:Concentration	3	0.212	0.071	1.926	0.1290
	Treatment:Carbon	3	1.602	0.534	14.582	3.62e-08
	Concentration:Carbon	9	1.500	0.167	4.551	3.55e-05
	Treatment:Concentration:Carbon	9	0.724	0.080	2.197	0.0267
	Residuals	121	4.431	0.037		

Supplemental Table S3. Mean viable counts[a] for Day 7 of respirometer experiments for all treatments.

	SE45 Respirometer[b]	E-37 Respirometer[c]	Community Respirometer[d]
No C	$1.34 \times 10^{5} \pm 1.38 \times 10^{5}$	$2.28 \times 10^{4} \pm 6.55 \times 10^{3}$	$6.63 \times 10^{5} \pm 2.57 \times 10^{5}$
NOM	$2.79 \times 10^{5} \pm 1.48 \times 10^{5}$	$2.17 \times 10^{5} \pm 6.41 \times 10^{4}$	$2.57 \times 10^{6} \pm 2.57 \times 10^{5}$
1 µM-C Casamino LOM	$3.97 \times 10^{5} \pm 5.42 \times 10^{4}$	$5.73 \times 10^{4} \pm 6.66 \times 10^{3}$	$6.37 \times 10^{5} \pm 7.02 \times 10^{4}$
1 µM-C Casamino mix	$4.30 \times 10^{5} \pm 2.17 \times 10^{4}$	$2.56 \times 10^{5} \pm 5.28 \times 10^{4}$	$1.51 \times 10^{6} \pm 1.11 \times 10^{5}$
4 µM-C Casamino LOM	$3.53 \times 10^{4} \pm 1.06 \times 10^{4}$	$3.33 \times 10^{5} \pm 4.07 \times 10^{5}$	$8.03 \times 10^{6} \pm 1.91 \times 10^{6}$
4 µM-C Casamino mix	$1.78 \times 10^{5} \pm 9.05 \times 10^{4}$	$3.30 \times 10^{5} \pm 4.00 \times 10^{4}$	$2.26 \times 10^{7} \pm 2.88 \times 10^{6}$
40 µM-C Casamino LOM	$1.49 \times 10^{5} \pm 5.82 \times 10^{4}$	$3.10 \times 10^{5} \pm 3.91 \times 10^{5}$	$2.15 \times 10^{7} \pm 2.40 \times 10^{6}$
40 µM-C Casamino mix	$4.71 \times 10^{5} \pm 1.21 \times 10^{5}$	$3.40 \times 10^{5} \pm 7.55 \times 10^{4}$	$3.27 \times 10^{6} \pm 4.62 \times 10^{5}$
400 µM-C Casamino LOM	$4.40 \times 10^{5} \pm 1.61 \times 10^{5}$	$2.93 \times 10^{5} \pm 4.73 \times 10^{4}$	$1.27 \times 10^{7} \pm 2.40 \times 10^{6}$
400 µM-C Casamino mix	$1.58 \times 10^{6} \pm 8.13 \times 10^{5}$	$7.53 \times 10^{5} \pm 3.21 \times 10^{4}$	$2.09 \times 10^{7} \pm 2.12 \times 10^{6}$
400 µM-C Acetate LOM	$1.07 \times 10^{7} \pm 3.96 \times 10^{5}$	$2.27 \times 10^{5} \pm 7.09 \times 10^{4}$	$9.87 \times 10^{6} \pm 9.29 \times 10^{5}$
400 µM-C Acetate mix	$8.31 \times 10^{6} \pm 1.08 \times 10^{6}$	$8.97 \times 10^{5} \pm 7.64 \times 10^{4}$	$1.74 \times 10^{7} \pm 2.29 \times 10^{6}$
400 µM-C Coumarate LOM	$2.55 \times 10^{6} \pm 4.72 \times 10^{5}$	$5.57 \times 10^{5} \pm 5.86 \times 10^{4}$	$1.45 \times 10^{6} \pm 2.12 \times 10^{5}$
400 µM-C Coumarate mix	$3.96 \times 10^{6} \pm 6.41 \times 10^{5}$	$7.47 \times 10^{5} \pm 6.11 \times 10^{4}$	$3.18 \times 10^{6} \pm 1.60 \times 10^{6}$

[a]The mean and one standard deviation are reported. No C and NOM CFU/mL was calculated from n=6, while the CFU/mL for the mix and LOM treatments was calculated from n=3.
[b]The seeding density for the SE45 respirometer experiments was 3.05×10^{4} CFU/mL ($\pm 7.97 \times 10^{3}$).
[d]The seeding density for the E-37 respirometer experiments was 1.43×10^{4} CFU/mL ($\pm 4.71 \times 10^{3}$).
[f]The seeding density for the community respirometer experiments was 5.13×10^{3} CFU/mL ($\pm 3.73 \times 10^{3}$).

Supplemental Table S4. Average amount of CO_2 (µg) respired by the final time (Day 7) point for each treatment.

Treatment	composite	LOM	NOM[a]	mix
SE45<br>400 µM-C Acetate	1153.63±364.83	963.72±415.24	189.91±51.34	826.51±221.18
SE45<br>1 µM-C Casamino Acids	73.39±120.38	-116.52±125.78	189.91±51.34	262.94±62.97
SE45<br>4 µM-C Casamino Acids	300.52±109.89	63.31±31.57	158.66±168.97	190.11±84.88
SE45<br>40 µM-C Casamino Acids	591.74±130.24	354.53±227.55	158.66±168.97	695.02±358.06
SE45<br>400 µM-C Casamino Acids	1283.08±76.02	1045.87±53.91	158.66±168.97	550.02±155.51
SE45<br>400 µM-C Coumarate	974.44±42.41	784.53±61.92	189.91±51.34	777.09±161.76
E-37<br>400 µM-C Acetate	750.16±127.13	426.02±147.20	324.12±175.10	3972.30±94.83
E-37<br>1 µM-C Casamino Acids	162.75±74.71	8.50±5.23	154.24±78.24	227.88±86.90
E-37<br>4 µM-C Casamino Acids	134.84±17.07	-19.41±72.83	154.24±78.24	32.69±16.46
E-37<br>40 µM-C Casamino Acids	158.12±83.59	3.882±39.58	154.24±78.24	141.60±34.70
E-37<br>400 µM-C Casamino Acids	522.59±241.11	198.45±134.40	324.12±175.10	2817.89±13.65
E-37<br>400 µM-C Coumarate	1706.55±17.47	1289.16±78.09	324.12±175.10	3473.37+122.56
Constructed Community<br>400 µM-C Acetate	838.13±49.20	525.05±15.70	313.08±33.90	612.50 ±14.20
Constructed Community<br>1 µM-C Casamino Acids	591.67±16.17	293.57±20.93	298.10±4.80	542.65±27.33
Constructed Community<br>4 µM-C Casamino Acids	1000.83±32.32	702.73±28.38	298.10±4.80	1085.36±28.65
Constructed Community<br>40 µM-C Casamino Acids	1395.32±18.69	1097.22±23.49	298.10±4.80	723.12±40.75
Constructed Community<br>400 µM-C Casamino Acids	429.31±39.80	116.23±6.91	313.08±33.90	257.37±24.35
Constructed Community<br>400 µM-C Coumarate	1368.75±85.47	1055.67±73.57	313.08±33.90	952.79±23.31

[a] Samples were processed in batch. Variation in NOM alone values represents run-to-run variation as these controls were included in every batch run.

Supplemental Table S5. ANOVA Tables for the three-way ANOVAs used to analyze the respiration data.

Test[a]	ANOVA Table	Df	Sum Sq	Mean Sq	F value	Pr(>F)
Accumulation Concentration[b]	Treatment	1	143017	143017	15.13	0.000315
	Concentration	3	3827240	1275747	134.95	< 2e-16
	Strain	2	1773619	886809	93.81	< 2e-16
	Treatment:Concentration	3	1653257	551086	58.30	7.17e-16
	Treatment:Strain	2	1784833	892417	94.40	< 2e-16
	Concentration:Strain	6	9157936	1526323	161.46	< 2e-16
	Treatment:Concentration:Strain	6	5517365	919561	97.28	< 2e-16
	Residuals	47	444303	9453		
Accumulation LOM Source (Carbon)[c]	Treatment	1	5012379	5012379	56.270	8.71e-09
	Carbon	2	2018650	1009325	11.331	0.000161
	Strain	2	18059959	9029980	101.373	2.75e-15
	Treatment:Carbon	2	722775	361387	4.057	0.026023
	Treatment:Strain	2	21359893	10679946	119.896	< 2e-16
	Carbon:Strain	4	1172539	293135	3.291	0.021667
	Treatment:Carbon:Strain	4	1423517	355879	3.995	0.008985
	Residuals	35	3117682	89077		
Rates Concentration[b]	Treatment	1	134.5	134.54	425.61	<2e-16
	Concentration	3	219.1	73.02	231.00	<2e-16
	Strain	2	108.7	54.36	171.96	<2e-16
	Treatment:Concentration	3	92.0	30.68	97.06	<2e-16
	Treatment:Strain	2	125.0	62.48	197.66	<2e-16
	Concentration:Strain	6	462.6	77.09	243.89	<2e-16
	Treatment:Concentration:Strain	6	291.5	48.59	153.71	<2e-16
	Residuals	47	14.9	0.32		
Rates LOM Source (Carbon)[c]	Treatment	2	1257.5	628.7	907.40	< 2e-16
	Carbon	2	88.4	44.2	63.77	1.02e-14
	Strain	2	645.5	322.8	465.82	< 2e-16
	Treatment:Carbon	4	72.2	18.0	26.03	7.06e-12
	Treatment:Strain	4	1357.0	339.2	489.60	< 2e-16
	Carbon:Strain	4	37.2	9.3	13.41	1.41e-07
	Treatment:Carbon:Strain	8	74.0	9.2	13.35	2.91e-10
	Residuals	52	36.0	0.7		

[a]Due to the unbalanced nature of the respirometer experimental design two three-way ANOVAs were used to analyze the differences between mix and composite in terms of CO_2 accumulation and production rates. The two ANOVA models used tested whether the independent variables of inoculum, treatment, and either LOM source or concentration interacted to affect CO_2 accumulation or production rates.

[b]The independent variables assessed in this ANOVA model were inoculum (or strain), concentration, and treatment.

[c]The independent variables assessed in this ANOVA model were inoculum (or strain), LOM source (or carbon), and treatment.

Table S6. Average Rate of CO_2 production (µg/h) for experiments shown in Figures 2 and 4.

Treatment	LOM	NOM[a]	MIX
SE45<br>400 µM-C Acetate	6.29±1.85	0.98±0.21	5.65±1.68
SE45<br>1 µM-C Casamino Acids	-1.09±0.80	0.98±0.21	1.46±0.03
SE45<br>4 µM-C Casamino Acids	0.84±0.08	1.99±0.41	1.61±0.51
SE45<br>40 µM-C Casamino Acids	2.90±0.44	1.99±0.41	695.02±358.06
SE45<br>400 µM-C Casamino Acids	6.38±0.07	1.99±0.41	3.44±0.71
SE45<br>400 µM-C Coumarate	6.06±0.29	0.98±0.21	7.68±2.06
E-37<br>400 µM-C Acetate	3.18±0.02	3.46±0.97	27.85±0.41
E-37<br>1 µM-C Casamino Acids	-0.26±0.27	2.05±0.23	2.57±0.77
E-37<br>4 µM-C Casamino Acids	-0.65±0.55	2.05±0.23	1.27±0.34
E-37<br>40 µM-C Casamino Acids	-0.44±0.44	2.05±0.23	2.34±0.08
E-37<br>400 µM-C Casamino Acids	1.74±1.07	3.46±0.97	20.66±0.15
E-37<br>400 µM-C Coumarate	8.40±0.73	3.46±0.97	23.64±0.81
Constructed Community<br>400 µM-C Acetate	4.69±0.27	3.35±0.29	5.80 ±0.11
Constructed Community<br>1 µM-C Casamino Acids	2.45±0.15	3.19±0.07	4.29±0.22
Constructed Community<br>4 µM-C Casamino Acids	6.29±0.29	3.19±0.07	9.14±0.12
Constructed Community<br>40 µM-C Casamino Acids	8.90±0.07	3.19±0.07	6.12±0.38
Constructed Community<br>400 µM-C Casamino Acids	1.66±0.17	3.35±0.29	3.21±0.35
Constructed Community<br>400 µM-C Coumarate	9.00±0.94	3.35±0.29	9.10±0.22

[a] Samples were processed in batch. Variation in NOM alone values represents run-to-run variation as these controls were included in every batch run.

Table S7. Probability values[a] for differences between respiration rates (µg/h) of LOM and mix.

	LOM Concentration[b]			
**LOM Source**	**1 µM-C**	**4 µM-C**	**40 µM-C**	**400 µM-C**
**Acetate**	Not Measured	Not Measured	Not Measured	SE45: $p < 0.50$<br>E-37: $\boldsymbol{p < 0.001}$<br>Community: $p < 0.20$
**Casamino Acids**	SE45: $\boldsymbol{p < 0.001}$<br>E-37: $\boldsymbol{p < 0.001}$<br>Community: $\boldsymbol{p < 0.001}$	SE45: $p < 0.15$<br>E-37: $\boldsymbol{p < 0.001}$<br>Community: $\boldsymbol{p < 0.001}$	SE45: $\boldsymbol{p < 0.001}$<br>E-37: $\boldsymbol{p < 0.001}$<br>Community: $p < 0.001$	SE45: $p < 0.001$<br>E-37: $\boldsymbol{p < 0.001}$<br>Community: $p < 0.10$
**Coumarate**	Not Measured	Not Measured	Not Measured	SE45: $\boldsymbol{p < 0.05}$<br>E-37: $\boldsymbol{p < 0.001}$<br>Community: $p < 0.95$

[a] Due to the unbalanced nature of the respirometer experimental design two three-way ANOVAs were used to analyze the differences between mix and LOM in terms of CO_2 production rates. The two ANOVA models used tested whether the independent variables of inoculum, treatment, and either LOM source or concentration interacted to affect CO_2 accumulation. As the 400 µM-C Casamino Acids accumulation was analyzed in both ANOVAs, the higher of the two resulting p-value from the post hoc test were used to determine significance. *p*-values are adjusted to correct for the false discovery rate using the Benjamini-Hochberg correction.

Supplemental Table S8. ANOVA Tables for each three-way ANOVA performed for the constructed community cell density by day.

Day	ANOVA Table	Df	Sum Sq	Mean Sq	F value	Pr(>F)
1	Treatment	1	1.185e+11	1.185e+11	0.017	0.89602
	Concentration	3	1.616e+16	5.386e+15	779.351	< 2e-16
	Carbon	3	5.357e+15	1.786e+15	258.377	< 2e-16
	Treatment:Concentration	3	8.469e+13	2.823e+13	4.085	0.00836
	Treatment:Carbon	3	4.262e+13	1.421e+13	2.056	0.10956
	Concentration:Carbon	9	1.266e+16	1.407e+15	203.509	< 2e-16
	Treatment:Concentration:Carbon	9	9.517e+13	1.057e+13	1.530	0.14447
	Residuals	124	8.570e+14	6.911e+12		
2	Treatment	1	4.666e+13	4.666e+13	2.004	0.160
	Concentration	3	1.809e+16	6.032e+15	259.014	<2e-16
	Carbon	3	3.011e+15	1.004e+15	43.106	<2e-16
	Treatment:Concentration	3	1.146e+14	3.819e+13	1.640	0.185
	Treatment:Carbon	3	4.151e+13	1.384e+13	0.594	0.620
	Concentration:Carbon	9	8.992e+15	9.991e+14	42.903	<2e-16
	Treatment:Concentration:Carbon	9	1.691e+14	1.879e+13	0.807	0.611
	Residuals	101	2.352e+15	2.329e+13		
4	Treatment	1	4.026e+10	4.026e+10	0.003	0.95598
	Concentration	3	8.482e+15	2.827e+15	214.882	< 2e-16
	Carbon	3	3.295e+15	1.098e+15	83.482	< 2e-16
	Treatment:Concentration	3	6.241e+13	2.080e+13	1.581	0.19732
	Treatment:Carbon	3	1.543e+14	5.142e+13	3.908	0.01045
	Concentration:Carbon	9	8.542e+15	9.491e+14	72.132	< 2e-16
	Treatment:Concentration:Carbon	9	3.956e+14	4.395e+13	3.340	0.00108
	Residuals	125	1.645e+15	1.316e+13		
7	Treatment	1	5.033e+13	5.033e+13	5.902	0.016597
	Concentration	3	4.782e+15	1.594e+15	186.894	< 2e-16
	Carbon	3	1.219e+15	4.063e+14	47.648	< 2e-16
	Treatment:Concentration	3	1.778e+14	5.925e+13	6.948	0.000236
	Treatment:Carbon	3	1.985e+13	6.617e+12	0.776	0.509620
	Concentration:Carbon	9	3.138e+15	3.487e+14	40.884	< 2e-16
	Treatment:Concentration:Carbon	9	8.561e+13	9.513e+12	1.115	0.357067
	Residuals	121	1.032e+15	8.528e+12		
10	Treatment	1	1.178e+14	1.178e+14	12.658	0.000532
	Concentration	3	6.171e+15	2.057e+15	221.110	< 2e-16
	Carbon	3	2.062e+15	6.874e+14	73.895	< 2e-16
	Treatment:Concentration	3	5.647e+14	1.882e+14	20.234	1.00e-10
	Treatment:Carbon	3	2.959e+14	9.865e+13	10.604	2.99e-06
	Concentration:Carbon	9	5.634e+15	6.260e+14	67.292	< 2e-16
	Treatment:Concentration:Carbon	9	6.062e+14	6.736e+13	7.241	2.14e-08
	Residuals	123	1.144e+15	9.303e+12		
14	Treatment	1	7.619e+13	7.619e+13	15.55	0.000144
	Concentration	3	4.007e+15	1.336e+15	272.65	< 2e-16
	Carbon	3	1.438e+15	4.795e+14	97.88	< 2e-16
	Treatment:Concentration	3	3.158e+14	1.053e+14	21.48	6.01e-11
	Treatment:Carbon	3	1.636e+14	5.453e+13	11.13	2.07e-06
	Concentration:Carbon	9	3.337e+15	3.708e+14	75.69	< 2e-16
	Treatment:Concentration:Carbon	9	4.453e+14	4.948e+13	10.10	4.33e-11
	Residuals	106	5.193e+14	4.899e+12		

Supplemental Table S9. ANOVA tables for each three-way ANOVA performed for constructed community alpha diversity estimates by day.

Day	ANOVA Table					
1		Df	Sum Sq	Mean Sq	F value	Pr(>F)
	Treatment	1	1.89	1.89	8.115	0.005141
	Concentration	3	110.94	36.98	158.782	< 2e-16
	Carbon	3	28.73	9.58	41.123	< 2e-16
	Treatment:Concentration	3	0.64	0.21	0.922	0.432564
	Treatment:Carbon	3	1.73	0.58	2.479	0.064284
	Concentration:Carbon	9	16.19	1.80	7.725	5.8e-09
	Treatment:Concentration:Carbon	9	8.51	0.95	4.062	0.000138
	Residuals	124	28.88	0.23		
2		Df	Sum Sq	Mean Sq	F value	Pr(>F)
	Treatment	1	0.39	0.39	1.037	0.310547
	Concentration	3	125.41	41.80	110.947	< 2e-16
	Carbon	3	13.83	4.61	12.235	4.71e-07
	Treatment:Concentration	3	1.60	0.53	1.418	0.240679
	Treatment:Carbon	3	0.58	0.19	0.516	0.671937
	Concentration:Carbon	9	13.47	1.50	3.973	0.000182
	Treatment:Concentration:Carbon	9	3.29	0.37	0.971	0.467301
	Residuals	122	45.97	0.38		
4		Df	Sum Sq	Mean Sq	F value	Pr(>F)
	Treatment	1	0.00	0.003	0.015	0.904
	Concentration	3	59.15	19.718	103.707	< 2e-16
	Carbon	3	7.13	2.377	12.500	3.39e-07
	Treatment:Concentration	3	4.75	1.583	8.328	4.34e-05
	Treatment:Carbon	3	1.05	0.348	1.832	0.145
	Concentration:Carbon	9	10.01	1.112	5.851	8.91e-07
	Treatment:Concentration:Carbon	9	9.34	1.037	5.456	2.67e-06
	Residuals	124	23.58	0.190		
7		Df	Sum Sq	Mean Sq	F value	Pr(>F)
	Treatment	1	0.37	0.365	3.344	0.0699
	Concentration	3	39.20	13.066	119.573	< 2e-16
	Carbon	3	5.25	1.752	16.030	7.74e-09
	Treatment:Concentration	3	1.19	0.398	3.639	0.0148
	Treatment:Carbon	3	1.04	0.348	3.186	0.0263
	Concentration:Carbon	9	6.13	0.681	6.232	3.36e-07
	Treatment:Concentration:Carbon	9	4.19	0.465	4.258	8.15e-05
	Residuals	121	13.22	0.109		
10		Df	Sum Sq	Mean Sq	F value	Pr(>F)
	Treatment	1	10.731	10.731	125.149	< 2e-16
	Concentration	3	30.257	10.086	117.623	< 2e-16
	Carbon	3	7.102	2.367	27.608	1.01e-13
	Treatment:Concentration	3	1.474	0.491	5.730	0.00105
	Treatment:Carbon	3	0.191	0.064	0.743	0.52814
	Concentration:Carbon	9	4.403	0.489	5.705	1.36e-06
	Treatment:Concentration:Carbon	9	3.389	0.377	4.391	5.46e-05
	Residuals	123	10.547	0.086		
14		Df	Sum Sq	Mean Sq	F value	Pr(>F)
	Treatment	1	19.789	19.789	215.291	< 2e-16
	Concentration	3	17.150	5.717	62.191	< 2e-16
	Carbon	3	25.118	8.373	91.088	< 2e-16
	Treatment:Concentration	3	5.414	1.805	19.635	1.88e-10
	Treatment:Carbon	3	1.483	0.494	5.379	0.001640
	Concentration:Carbon	9	10.376	1.153	12.543	6.26e-14
	Treatment:Concentration:Carbon	9	2.833	0.315	3.425	0.000867
	Residuals	122	11.214	0.092		

Supplemental Figures

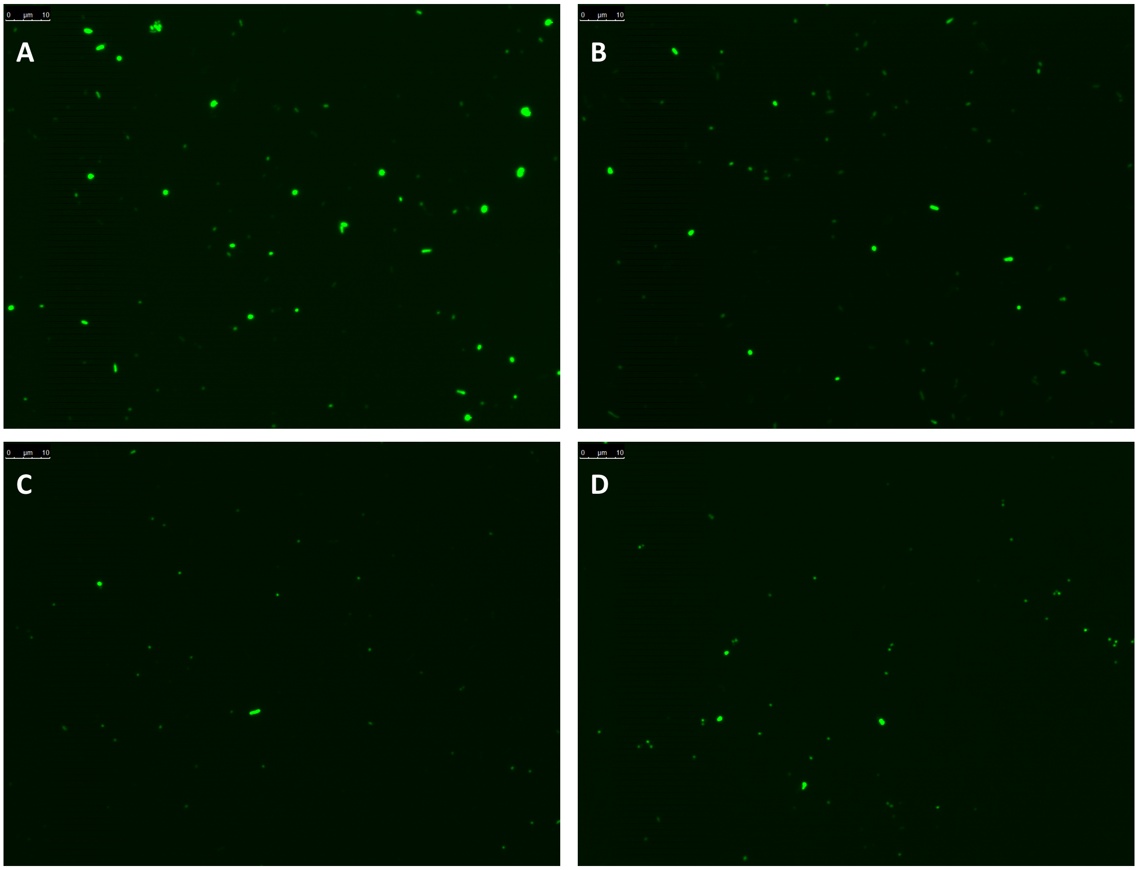

Supplemental Figure 1. Microscopic images of representative constructed community cultures grown on (A & B) 400 uM casamino acids + NOM on Day 3; replicate cultures and (C & D) on only 400 uM casamino acids on Day 6. Cultures were stained with DAPI and collected on a 0.22- µm polycarbonate filter. A scale bar is provided in the upper left-hand corner of each image.

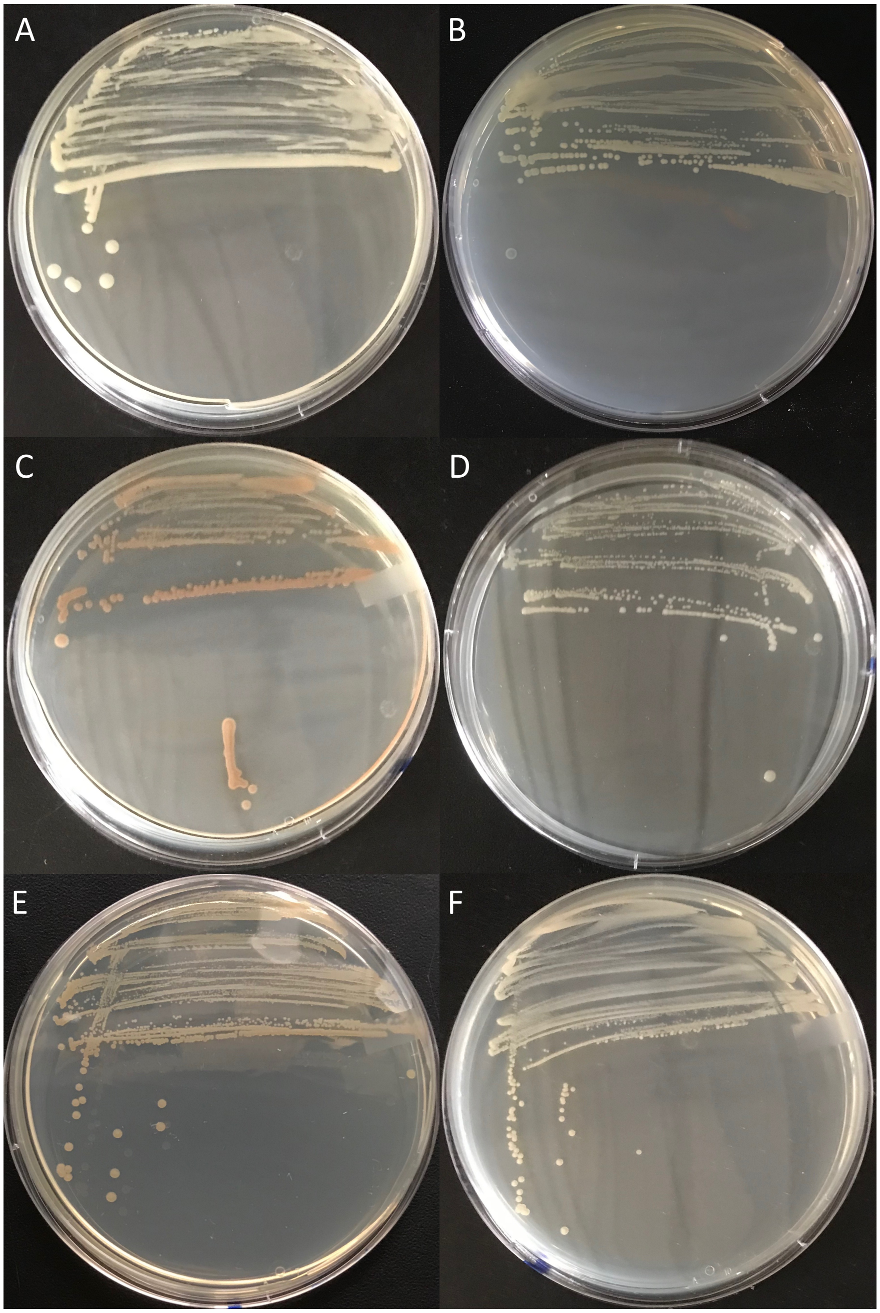

Supplemental Figure 2. Colony morphologies of the bacterial isolates used in the constructed community. A. *Citreicella* sp. SE45 B. *Phaeobacter* sp. Y4I C. *Roseovarius nubinhibens* ISM D. *Sagittula stellata* E-37 E. *Sulfitobacter* sp. EE-36 F. *Sulfitobacter* sp. NAS-14.1

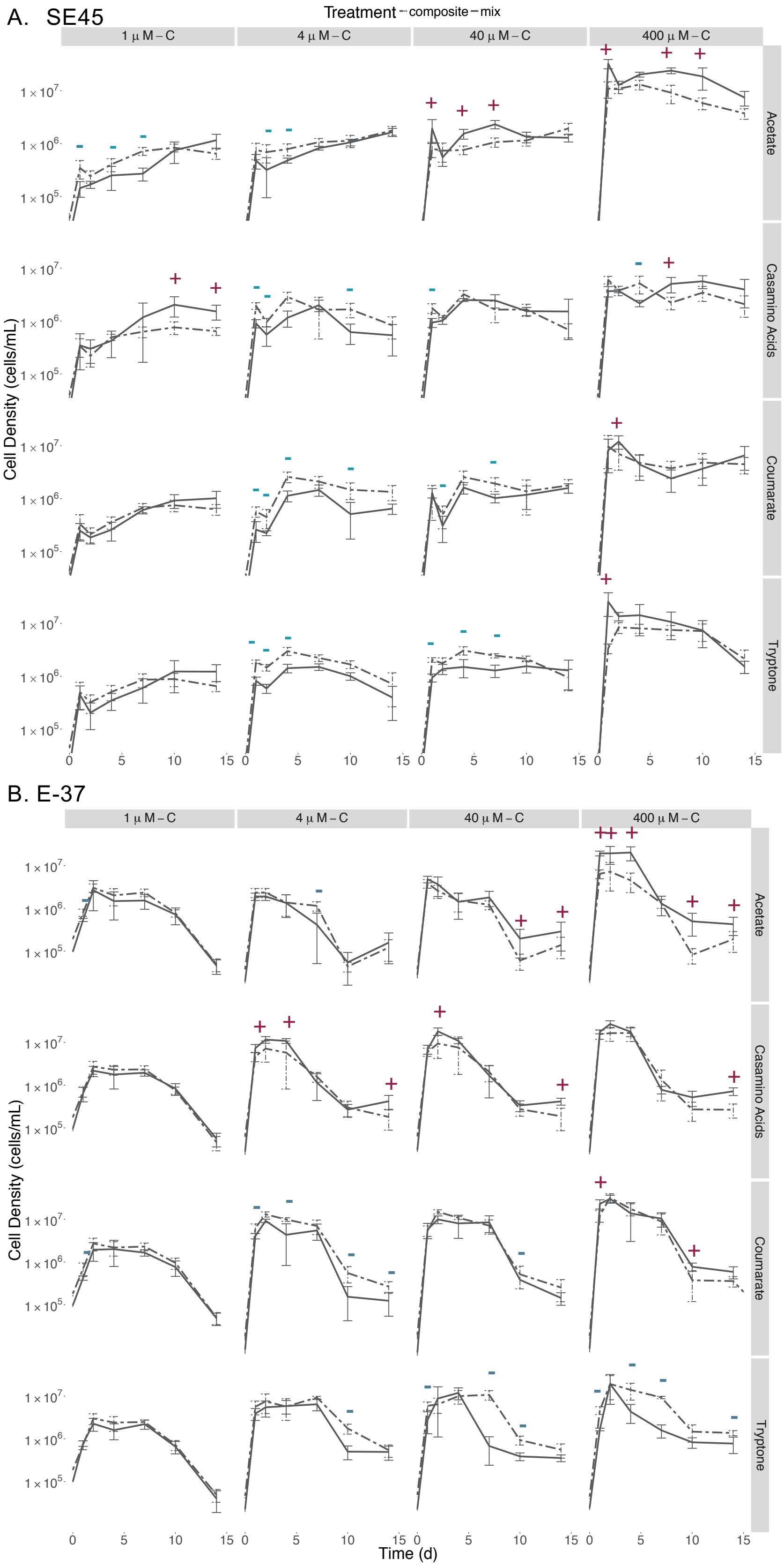
A. SE45
Treatment – composite – mix
1 µ M – C
4 µ M – C
40 µ M – C
400 µ M – C
Acetate
Casamino Acids
Coumarate
Tryptone
1 × 10^5
1 × 10^6
1 × 10^7
Cell Density (cells/mL)
0
5
10
15
B. E-37
Time (d)

Supplemental Figure S3. Viable counts for monocultures of (A) SE45 and (B) E-37 in composite (dashed line) and mix (black line) treatments.

Points represent the mean (n=3-5); error bars represent one standard deviation from the mean. Red plus signs indicate a significant synergistic interactive effect ($p < 0.05$), blue minus signs indicate a significant antagonistic interactive effect ($p < 0.05$). Seeding densities for SE45 and E-37 and six-member constructed community were 1.51 x 10^4 CFU/mL (±5.1 x 10^3) and 4.23 x 10^4 CFU/mL (±9 x 10^3). The composite treatment is the sum of results of the LOM and NOM treatments.

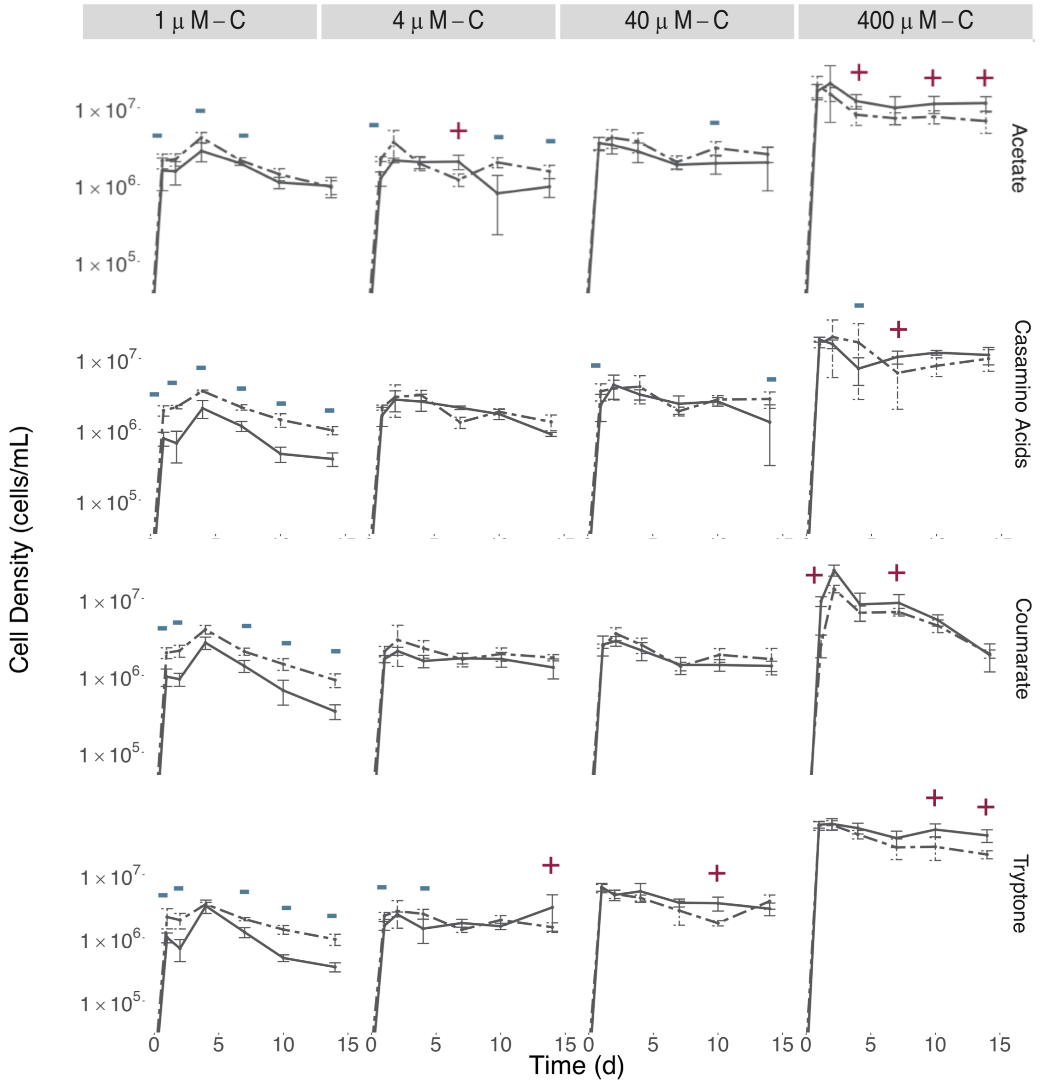

Supplemental Figure S4. Viable counts for constructed communities in composite (dashed and mix (black line) treatments.

Points represent the mean (n=3-5); error bars represent one standard deviation from the mean. Red plus signs indicate a significant synergistic interactive effect ($p < 0.05$), blue minus signs indicate a significant antagonistic interactive effect ($p < 0.05$). Seeding densities for six-member constructed community was 7.01 x 10^3 CFU/mL (±2.6 x 10^3), respectively. The composite treatment is the sum of results of the LOM and NOM treatments.

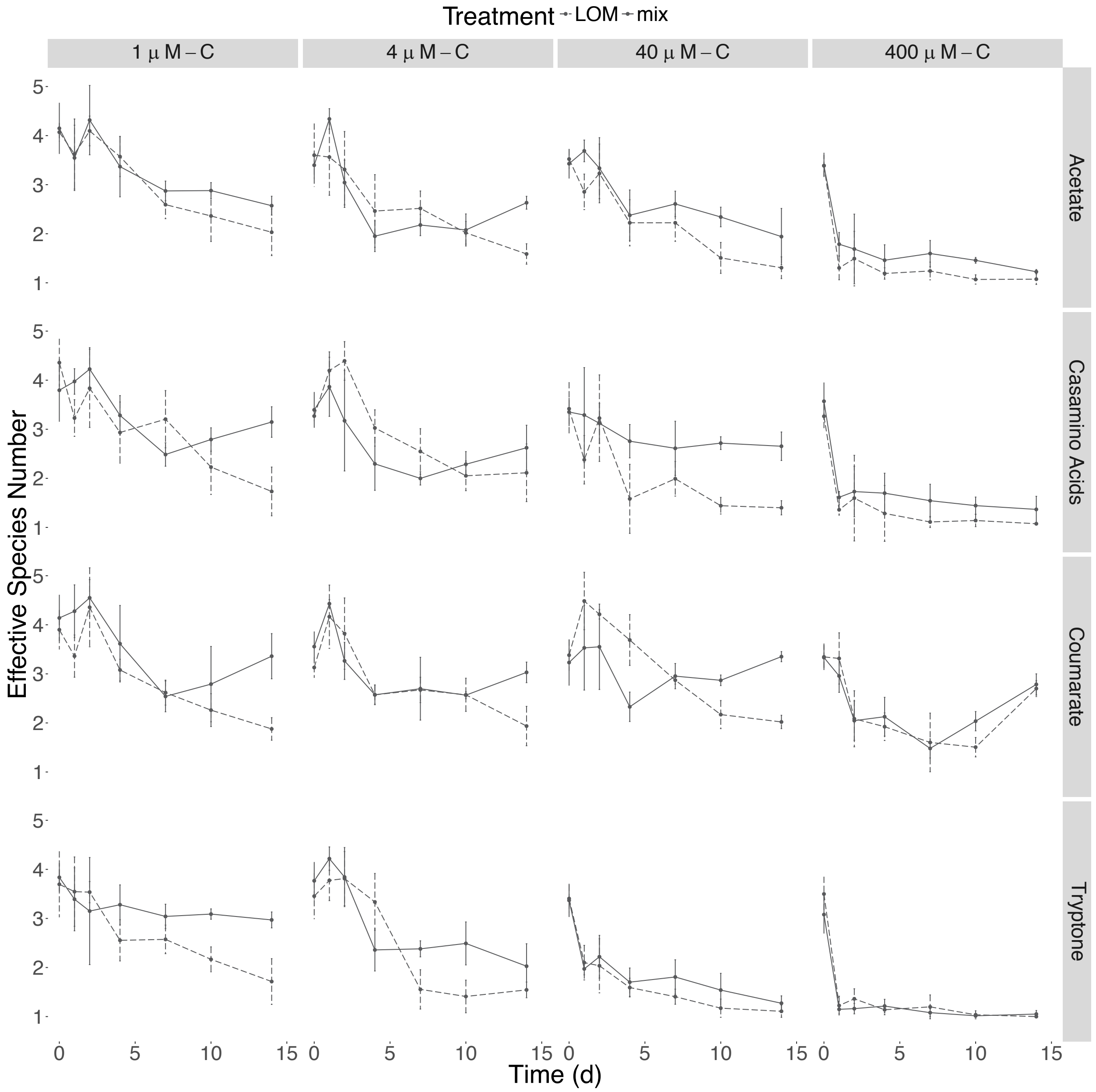

Supplemental Figure S5. Alpha diversity of constructed community under mixed substrate conditions.
Shannon entropy was calculated for each replicate at each time point. Shannon entropy was converted to Hill numbers or effective species number. Points represent the average of 3-5 replicates, and error bars represent one standard deviation from the mean. Dashed lines represent the LOM cultures while the solid lines represent the mixed carbon cultures.

A.

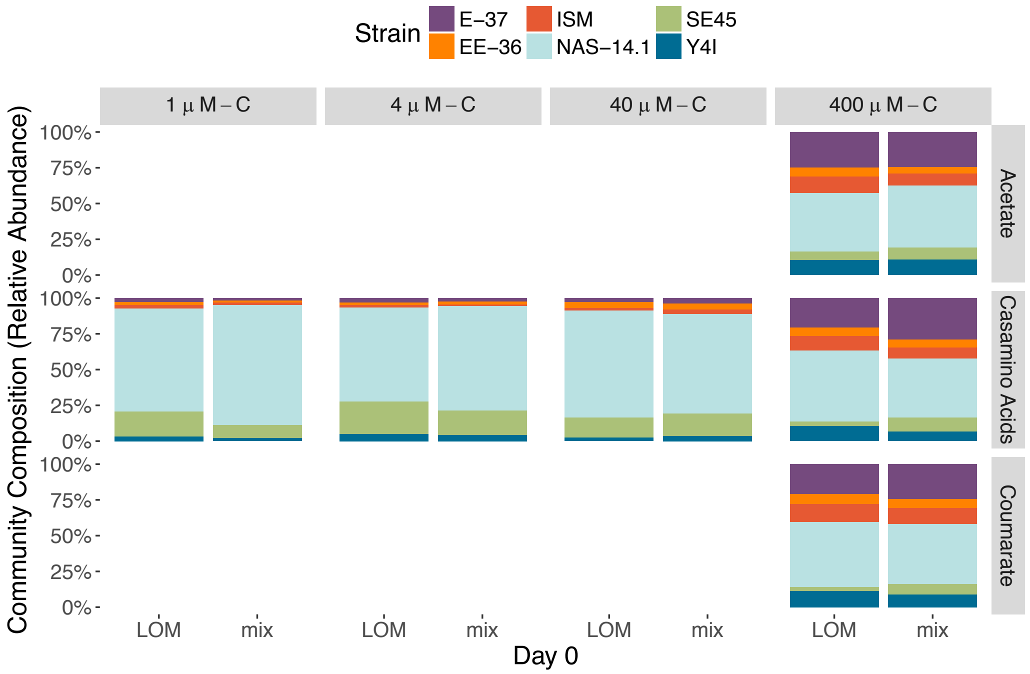
Strain
E−37
ISM
SE45
EE−36
NAS−14.1
Y4I
1 µ M − C
4 µ M − C
40 µ M − C
400 µ M − C
Acetate
Casamino Acids
Coumarate
Community Composition (Relative Abundance)
100%
75%
50%
25%
0%
LOM
mix
Day 0

B.

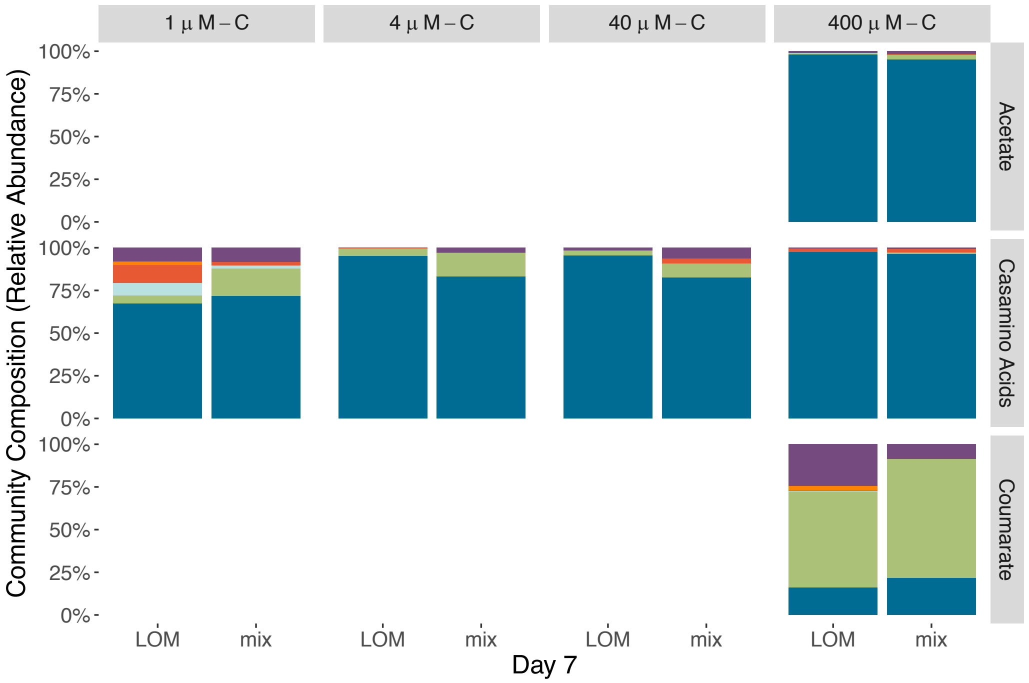
1 µ M − C
4 µ M − C
40 µ M − C
400 µ M − C
Acetate
Casamino Acids
Coumarate
Community Composition (Relative Abundance)
100%
75%
50%
25%
0%
LOM
mix
Day 7

C.

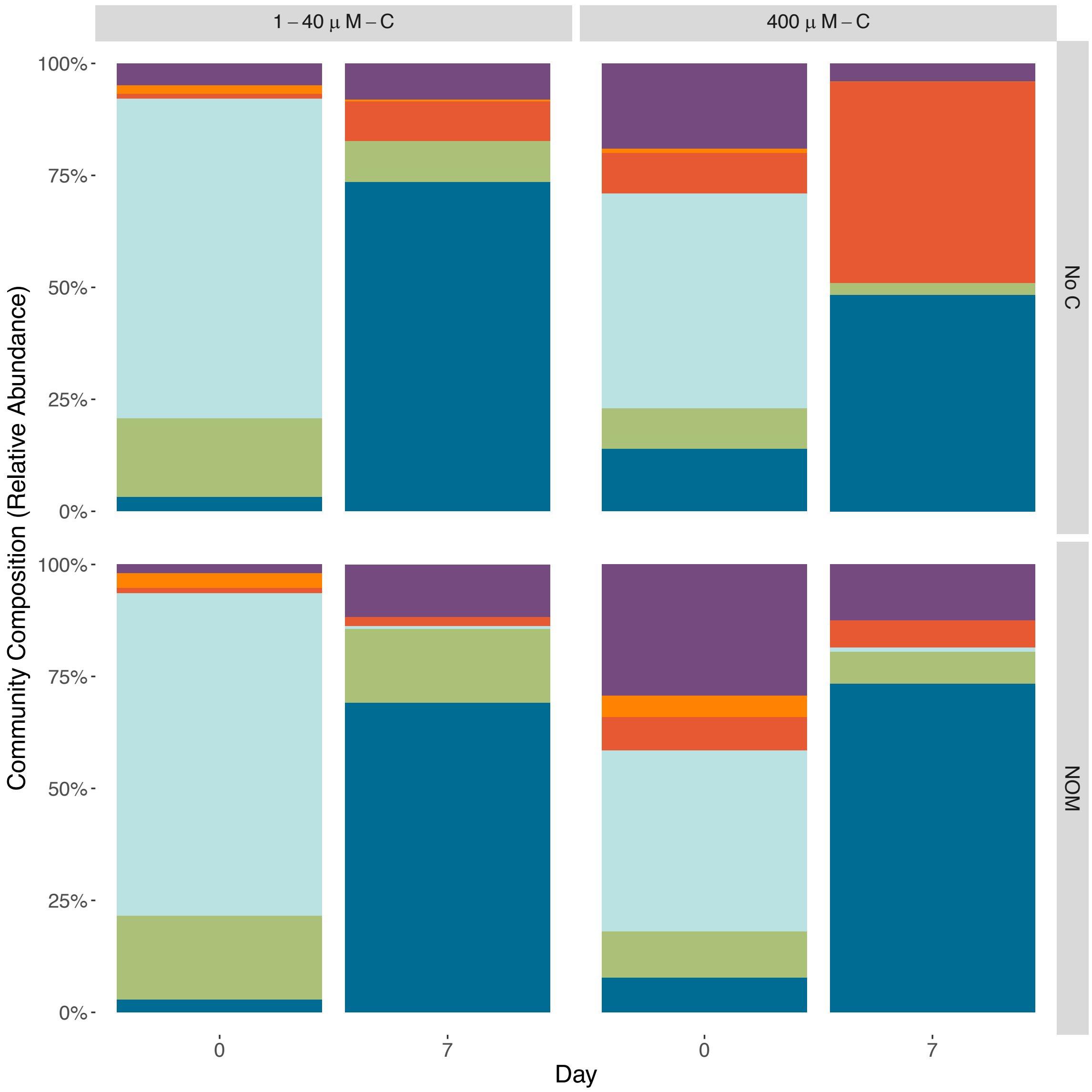

Supplemental Figure S6. The community composition of the respirometer incubations for the LOM and mix treatments at Day 0 (A) and Day 7. (B) The community composition for the NOM and No C treatments are present in **panel C**. The NOM and No C treatments for the first incubation which included the low concentrations of Casamino Acids (1, 4, and 40 uM) are in the left panel while the community composition for the NOM and No C treatments for the high concentrations of labile carbon are in the right panel.

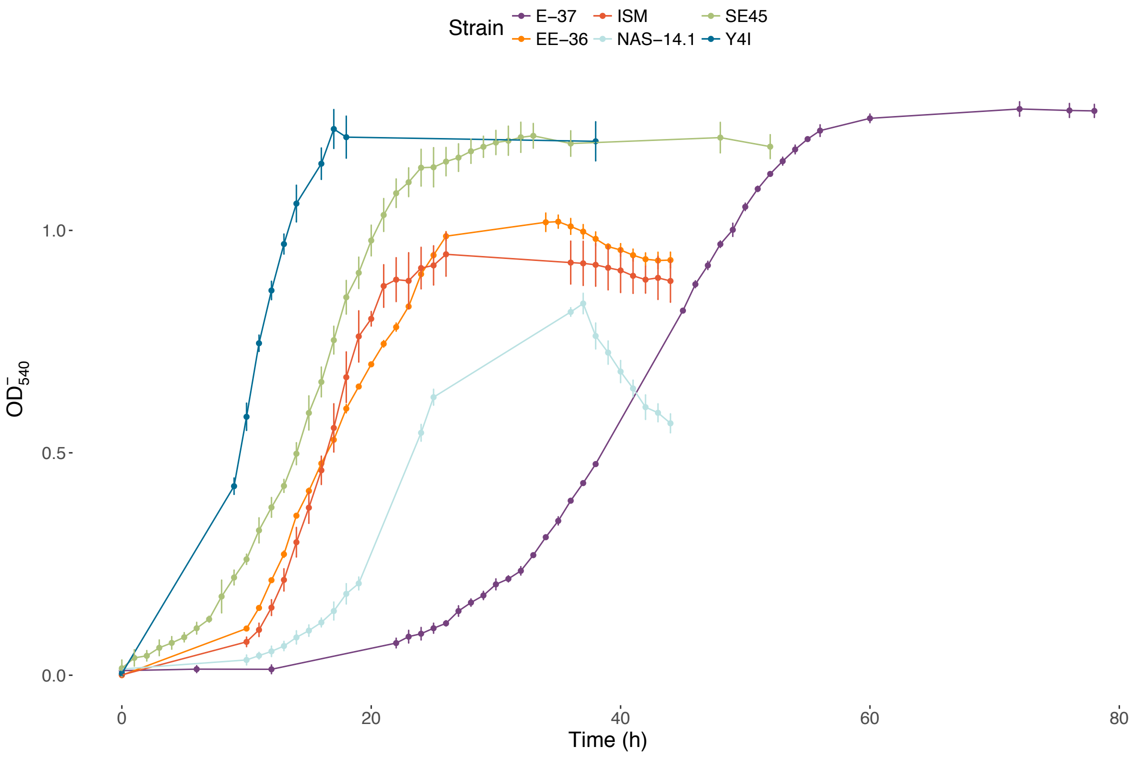

Supplemental Figure S7 Growth curves of the strains used in this study when grown as monocultures in minimal media supplemented with 2 mM-C tryptone. Points represent the mean of three biological replicates and error bars represent one standard deviation from the mean.
